# Identification of *BRCA1*:c.5470_5477del as a Founder Mutation in Chinese Ovarian Cancer Patients

**DOI:** 10.3389/fonc.2021.655709

**Published:** 2021-05-11

**Authors:** Jun Li, Sile Han, Cuiyun Zhang, Yanlin Luo, Li Wang, Ping Wang, Yi Wang, Qingxin Xia, Xiaoyan Wang, Bing Wei, Jie Ma, Hongle Li, Yongjun Guo

**Affiliations:** ^1^ Department of Molecular Pathology, The Affiliated Cancer Hospital of Zhengzhou University and Henan Cancer Hospital, Zhengzhou, China; ^2^ Henan Key Laboratory of Molecular Pathology, Zhengzhou, China; ^3^ Department of Gynecologic Oncology, The Affiliated Cancer Hospital of Zhengzhou University and Henan Cancer Hospital, Zhengzhou, China; ^4^ Department of Pathophysiology, School of Basic Medical Science, Zhengzhou University, Zhengzhou, China; ^5^ Department of Pathology, The Affiliated Cancer Hospital of Zhengzhou University and Henan Cancer Hospital, Zhengzhou, China

**Keywords:** *BRCA1/2*, haplotype analysis, ovarian cancer, founder mutation, Chinese

## Abstract

Predisposition of germline *BRCA1/2* mutations (*gBRCA^MUT^*) increases the risk of breast and ovarian cancer in females, but the mutation prevalence and spectrum are highly ethnicity-specific with different recurrent mutations being reported in different populations. Hereby, we performed hybridization-based target sequencing of *BRCA1/2* in 530 ovarian cancer patients from Henan, the central region of China, followed by haplotype analysis of six short tandem repeat (STR) markers in the patients with recurrent mutations to determine their founder effect. About 28.3% (150/530) of the OC patients in our cohort harbored *gBRCA^MUT^*; of the 151 mutations, 117 in *BRCA1* and 34 in *BRCA2*, identified in this study, *BRCA1*:c.5470_5477del, c.981_982del, and c.4065_4068del are the top three mutants, recurrently detected in eight, seven, and six independent patients respectively. Haplotype analysis identified a region of 0.6 MB genomic length covering *BRCA1* highly conserved across all eight carriers of *BRCA1*:c.5470_5477del, but not c.981_982del, suggesting a consequence of founder effect. Retrospective analysis in a subgroup of serous ovarian cancer patients revealed *gBRCA^MUT^* status was not associated with the progression-free survival (PFS); instead, an expression of Ki-67% ≥50% was associated with a shorter PFS (*p* = 0.041). In conclusion, patients with pathogenic or likely pathogenic *gBRCA^MUT^* account for 28.3% of the OC cases from Henan, and *BRCA1*:c.5470_5477del, the most frequently detected mutation in Henan patients, is a founder mutation in the population.

## Introduction

Ovarian cancer (OC) is the seventh commonest cancer in women, with a global morbidity and mortality rate of 6.6 and 3.9 per 100,000 population respectively, and the incidence has been increasing moderately over the last decade (1, 2). OC usually doesn’t cause noticeable symptoms and there’s no effective screening method; therefore, many patients are not diagnosed until the late stage of the disease, resulting in a relatively high mortality rate (3, 4). Several factors have been associated with the risk of developing OC, *e.g.*, endometriosis, family history, inherited genetic aberrations, *etc.*; particularly, 39–44% of women who inherited a pathogenic mutation in Breast Cancer 1 *(BRCA1*) and 11–17% of women with a pathogenic mutation in Breast Cancer 2 (*BRCA2*) will develop OC by 70–80 years of age (5, 6). *BRCA1* and *BRCA2*, located on 17q21 and 13q13 respectively, are well-established tumor suppressor genes that their protein products are of paramount importance in maintaining the genomic stability and integrity by facilitating error-free homologous recombination repair (HRR) of DNA double strand breaks (DSBs). Multiple studies have revealed that mutations in *BRCA1* and *BRCA2* are prevalent throughout the whole coding region and the flanking splice sites without any hot spots (7, 8); meanwhile, some mutations have been reported in specific populations with relatively high frequencies as a consequence of founder effect, *i.e.*, the 185delAG in Ashkenazi Jews (9), the 2804delAA in Dutch (10), and E881X in south Africans (11) *etc.* Khoo et al. first reported the *BRCA1*:c.1081del as a founder mutation in southern Chinese OC patients in 2002 (12). However, other groups to investigate the prevalence of germline *BRCA1/2* mutations (*gBRCA1/2^MUT^*) in Chinese OC patients revealed different mutations, *i.e., BRCA1:*c.5470_5477del and c.981_982del *etc.* (7, 13) Previous dispute, probably caused by different places of origin of the patients, inspired us to identify the *BRCA1/2* founder mutations in Henan OC patients; this is because Henan, located in the middle of China with over 100 million population mainly composed of Han Chinese, is the origin of Chinese civilization.

## Material and Methods

### Study Population

Patients diagnosed as ovarian cancer and referred to *BRCA1/2* mutation test in Henan Cancer Hospital from March 2018 to May 2020 were included in this study. Clinical information of these patients was retrieved from their medical records, including disease on-set age, location of the primary lesion, family history, immunohistochemistry (IHC) staining, treatment strategy, and response. Family history was defined as at least one first- or second-degree relative has been diagnosed as breast, ovarian cancer, prostate cancer or pancreatic cancer. Progression of the disease was evaluated according to Response Evaluation Criteria in Solid Tumors (RECIST) 1.1 criteria. The study was approved by the Ethics Committee of Henan Cancer Hospital and written informed consent was obtained from all patients.

### DNA Extraction and BRCA Mutation Detection

Genomic DNA was extracted from 500 µl peripheral-blood using QIAamp DNA isolation kit (QIAGEN, Germany) according to the manufacturer’s instructions. DNA concentration was determined by using the Qubit dsDNA HS assay (Life Technologies, the U.S.), and purity was evaluated with NanoDrop 2000 UV-Vis Spectrophotometer (Thermo Scientific, the U.S.) by measuring the ratio of absorbance at 260 and 280 nm. A total of 200 ng genomic DNA was used for library construction. Briefly, the harvested DNA was first sheared to size of approximately 300 bp by using the Bioruptor sonication device (Diagenode, U.S.A.); then the fragmented DNA was blunt-end-repaired and A-tailed to ligate with adapters, followed by PCR amplification and purification. Target-enriched library was prepared by using the cancer susceptible gene detection kit according to the instructions from the manufacturer (Novogene, China), covering a 0.26 Mb genomic region of 45 breast/ovarian cancer related genes. The enriched library was then processed for sequencing on a NextSeq550 sequencer (Illumina, the U.S.) generating paired-end reads of 150 bp to a targeted coverage of >500 unique reads.

### Assessment of Variants’ Pathogenicity

Pathogenicity of the variants detected in *BRCA1/2* of this study was evaluated according to the American College of Medical Genetics and Genomics (ACMG) guideline (14) and Evidence-based Network for the Interpretation of Germline Mutant Alleles (ENIGMA) criteria (v 2.5.1). Classification of the variants was performed by two clinical geneticists independently. Annotations of the variants followed the Human Genome Variant Society (HGVS) recommendations (15).

### Genotype Haplotype Analysis

The individuals with recurrent mutations and from irrelevant families were genotyped at six different polymorphic short tandem repeats (STR) loci adjacent to *BRCA1* on chromosome17, including D17S951, 17S1789, D17S846, D17S1818, D17S1327, and D17S1320. Sequences of the primers used in this study were obtained from the UCSC genome browser (http://www.genome.ucsc.edu/). PCR reaction was performed using fluorescently end-labeled primers with (**Supplementary Table 1**) the following program: 95°C for 3 min, followed by 10 cycles of 94°C for 30 s, 60°C for 30 s, and 72°C for 30 s, then 35 cycles of 94°C for 30 s, 55°C for 30 s and 72°C for 30 s, finally end with 72°C for 5 min. The amplicon was processed for size fractionation on a 3730xl Genetic Analyzer (Applied Biosystems, the U.S.) and analyzed using Genemapper™ software (Thermo Fisher Scientific, the U.S.) by Sangon Biotech. (Sangon, China).

### Statistical Analysis

A chi-square test was used to determine the statistical significance for categorial variables. Disease on-set age distribution of the patients was compared by using a log-rank test stratified according to their mutation status. Median progression-free survival of the patients was calculated with the Kaplan–Meier method and compared using the log-rank test. A p-value of <0.05 was considered as significant.

### Data Deposition

According to the Management of Human Genetic Resources in the People’s Republic of China, sequencing data related to this study is available from the corresponding author upon reasonable request.

## Results

### Patient Characteristics and the Association With BRCA Germline Mutation

In total, 530 patients were included in this study. Individuals of <40 years old at diagnosis accounted for 7.92% (42/530) *versus* 92.08% (488/530) of the patients ≥40 years old. Only 17.74% (94/530) of the patients were diagnosed at earlier stages (53 in stage-1 and 41 in stage-2), and majority presented with advanced disease (54.91% in stage-3 and 16.42% in stage-4). Breast cancer was observed in 4.53% (24/530) of the patients, in which 14 were *gBRCA1/2^MUT^* and 10 were *gBRCA1/2^WT^* carriers; other concurrent cancers were rare in this cohort. Serous ovarian cancer was the dominant pathological subtype, accounting for 80.94% (429/530) of the patients, followed by clear cell, endometrioid, and mucinous carcinoma. There were 55 patients referred from other hospitals, and their diagnosis was only recorded as ovarian cancer without clear subtyping. About 9.43% (50/530) of the patients in our study were recorded with family history (**Table 1**).

**Table 1:** Clinical characteristics of 530 Henan ovarian cancer patients.

Characteristics		cases/percentage					
		ALL (n = 530)	BRCA^WT^ (n = 380)	BRCA^MUT^ (n = 150*)	p-value	BRCA1^MUT^ (n = 117)	BRCA2^MUT^ (n = 34)
*Disease on-set age*	<40 years old	42(7.92%)	35(9.21%)	7(4.67%)	ns	7(5.98%)	0
	≥40 years old	488(92.08%)	345(90.79%)	143(95.33%)		110(94.02%)	34(100.00%)
*Stage*	I	53(10.00%)	45(11.84%)	8(5.33%)	ns	5(4.27%)	3(8.82%)
	II	41(7.74%)	25(6.58%)	16(10.67%)		13(11.11%)	4(11.76%)
	III	291(54.91%)	211(55.53%)	80(53.33%)		62(52.99%)	18(52.94%)
	IV	87(16.42%)	58(15.26%)	29(19.33%)		24(20.51%)	5(14.71%)
	unknown	58(10.94%)	41(10.79%)	17(11.33%)		13(11.11%)	4(11.76%)
*Metastasis*	Breast cancer	24(4.53%)	10(2.63%)	14(9.33%)	0.002	12(10.26%)	2(5.88%)
	Lung cancer	1(0.19%)	0	1(0.67%)	ns	0	1(2.94%)
	Thyroid cancer	4(0.75%)	4(1.05%)	0		0	0
	Esophagus cancer	2(0.38%)	1(0.26%)	1(0.67%)		1(0.85%)	0
	Colon cancer	1(0.19%)	1(0.26%)	0		0	0
	Acoustic	1(0.19%)	1(0.26%)	0		0	0
*Subtype*	Serous	429(80.94%)	294(77.37%)	135(90.00%)	0.000	110(94.02%)	26(76.47%)
	Mucinous	9(1.70%)	9(2.37%)	0		0	0
	Endometrioid	19(3.58%)	19(5.00%)	0		0	0
	Clear cell	18(3.40%)	18(4.74%)	0		0	0
	Unspecified	55(10.38%)	40(10.53%)	15(10.00%)		7(5.98%)	8(23.53%)
*Family history*	Yes	50(9.43%)	17(4.47%)	33(22.00%)	0.000	29(24.79%)	5(14.71%)
	No	480(90.57%)	363(95.53%)	117(78.00%)		88(75.21%)	29(85.29%)

*One patient carried both BRCA1 mutation and BRCA2 mutation. WT, wild-type; MUT, mutation; NS, not significant.

**Figure 1:**
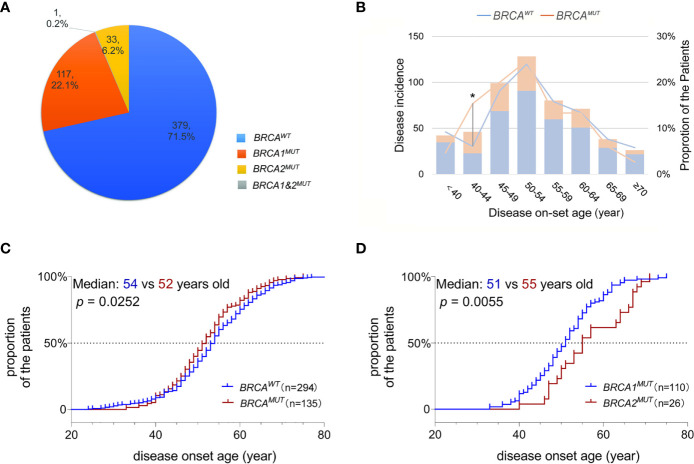
*BRCA* mutations associated with disease on-set age of OC. **(A)** A pie plot shows the number and proportion of *BRCA1* and *BRCA2* germline mutation carriers in 530 Henan OC patients. WT, wild-type; MUT, mutation. **(B)** Incidence (left Y-axis) and proportion of the patients developed serous OC (right Y-axis) at defined age (X-axis) in *BRCA^MUT^* (orange) and *BRCA^WT^* (blue) carriers. A chi-square test was used to determine whether the frequency of OC incidence is different between *BRCA^MUT^* and *BRCA^WT^* carriers of 40–44 years old. *p < 0.05. **(C)** Comparison of the disease on-set age of serous OC between *BRCA^MUT^ versus BRCA^WT^* carriers, and *BRCA1^MUT^ versus BRCA2^MUT^* carriers **(D)** by plotting the cumulative incidence curve. A log-ranked test was used to compare the difference in disease on-set age between different groups, and a *p*-value of < 0.05 was considered as significant.

In total, pathogenic or likely pathogenic *gBRCA1/2^MUT^* were identified in 28.3% (150/530) of the OC patients in our cohort; 117 patients carried *BRCA1^MUT^* and 33 carried *BRCA2^MUT^* and one patient carried both (**Figure 1A**). Although the disease incidence in both *BRCA^WT^* and *BRCA^MUT^* patients peak at 50–54 years old, *BRCA^MUT^* carriers had a significantly higher likelihood of developing OC between the age of 40 and 44 as compared to the *BRCA^WT^* carriers (*p* = 0.019, **Figure 1B**). Generally, the disease on-set age was slightly earlier in *BRCA^MUT^* carriers as compared to *BRCA^WT^* carriers (median: 52 *vs* 54 years old, *p* = 0.0252, **Figure 1C**), and in *BRCA1^MUT^* carriers *versus BRCA2^MUT^* carriers (median: 51 *vs* 55 years old, *p* = 0.0055, **Figure 1D**). Among the 42 patients presenting the disease before 40, seven patients harbored *BRCA1^MUT^* and the other 35 were *BRCA^WT^*, suggesting other unclarified risk factors contributing to their early disease on-set. Not surprisingly, *BRCA^MUT^* carriers more often developed breast cancers than other cancers (*p* = 0.002); *BRCA^MUT^* predominantly present in serous ovarian cancers rather than other subtypes (*p* = 0.000), and patients with a family showed a higher likelihood to carry *BRCA^MUT^* than the ones without (*p* = 0.000; **Table 1**).

### The Founder Effect of BRCA1:c.5470_5477del in Henan OC Patients

**Figure 2:**
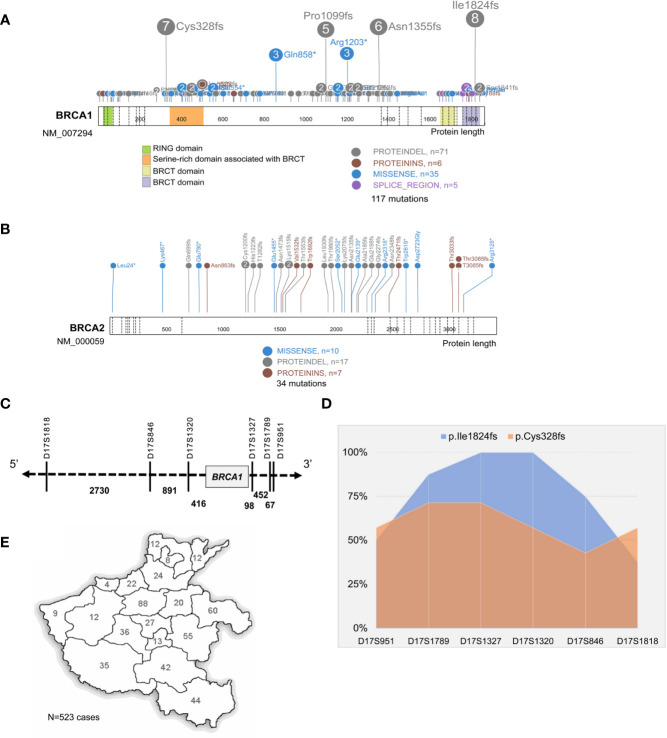
Identification of *BRCA1*:c.5470_5477del as the founder mutation in Henan OC patients. Protein paint shows the pathogenic and likely pathogenic mutations detected in *BRCA1* ***(*A*)*** and *BRCA2* **(B)** from 530 Henan OC patients. **(C)** A schematic diagram illustrates the genomic location of *BRCA1* and its flanking STR markers selected for haplotype analysis. **(D)** Haplotype analysis of six STR markers across 8 *BRCA1*:c.5470_5477del (p.lle1824fs) carriers (blue) and seven *BRCA1*:c.981_982del (p.Cys328fs) carriers (orange). STR loci are indicated on x-axis and the proportion of patients sharing the same allele is indicated on Y-axis. **(E)** A map shows the places of origin of the patients in this OC cohort.

Of the 151 mutations identified in our study, 117 were in *BRCA1* and 34 in *BRCA2*; frameshift is the dominant subtype (66.9%,101/151), followed by missense (33.1%, 50/151); six mutations were located on the canonical splice sites, and an intronic conversion of A>G at *BRCA1*:c.213_12, creating a novel 3′ acceptor splicing sites, was also identified (**Figures 2A, B**). About 21.9% (33/151) of the detected mutations have not been reported in either Clinvar or *BRCA* exchange database (accessed in Jan. 2020), suggesting a different *BRCA* mutation spectrum in Chinese as compared to other populations. *BRCA1*:c.5470_5477del was the most recurrently detected mutation in our cohort, presenting in eight independent individuals; followed by *BRCA1*:c.981_982del and *BRCA1*:c.4065_4068del. In total, the top three mutants accounted for 13.9% (21/151) of all the *BRCA* mutations detected in this study.

Then we performed haplotype analysis in the patients harboring *BRCA1*: c.5470_5477del by investigating six highly polymorphic STR markers flanking *BRCA1* on chromosome17 (**Figure 2C**); seven other patients, sharing the *BRCA1*:c.981_982del mutation, were included for comparison. The results showed there was a highly conservative region, with a genomic length of 0.6 MB covering *BRCA1*, shared by all eight unrelated *BRCA1*:c.5470_5477del carriers, and the conservation gradually descend toward both sides. Meanwhile, this region was only partially shared by the *BRCA1*:c.981_982del carriers (**Figure 2D** and **S Figure 1**). Collectively, our data support *BRCA1*:c.981_982del as the suspected founder mutation in Henan OC patients since 98.7% (523/530) of the patients in this cohort are from different regions of Henan province (**Figure 2E**).

### Ki-67 Expression, but Not gBRCA^MUT^ Status, Associated With the PFS of Serous OC Patients

**Figure 3:**
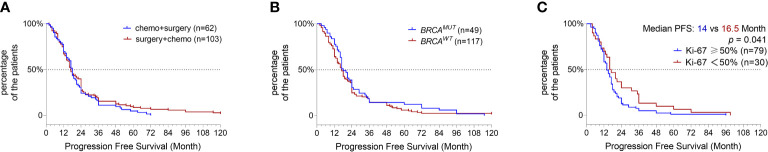
Progression-free survival (PFS) analysis. Kaplan–Meier plot shows the comparison of PFS in serous OC patients primarily treated by surgery *versus* adjuvant chemotherapy **(A)**, *BRCA^MUT^* carriers versus *BRCA^WT^* carriers **(B)**, and the patients with high ki-67 expression of ≥50% *versus <*50% **(C)**.

To prevent the confounding influence from patients with other subtypes of ovarian cancer, we focused our survival analysis on serous cancer only. In total, 165 serous OC patients at stage-3 or -4 disease with continuous follow-up data were investigated for their progression-free survival (PFS), and 103 patients were subjected to surgery resection followed by chemotherapy and the other 62 received adjuvant chemotherapy in advance to surgery. The analysis revealed neither the order of chemotherapy and surgery nor the *gBRCA^MUT^* status influenced the PFS of patients (**Figures 3A, B**); instead, the patients with a high Ki-67 expression of ≥50% nuclear staining in the FFPE sections showed a significantly shorter PFS (**Figure 3C**, *p* = 0.041), suggesting as a valuable prognostic predictor (Hazard ratio = 1.557 with 95% CI of 1.018–2.379). We didn’t observe any evident difference in the PFS between the patients with *BRCA1*:c.5470_5477del founder mutation and the ones with other *gBRCA^MUT^* (data not shown).

## Discussion

In this study, we identified 28.3% (150/530) of the OC patients in this Henan cohort as *gBRCA^MUT^* carriers, including one with concurrent mutations in both *BRCA1* and *BRCA2*. Haplotype analysis revealed a region of 0.6 MB genomic length spanning *BRCA1* highly conserved across all the independent carriers of *BRCA1*:c.5470_5477del, supporting it as a founder mutation in Henan population. Survival analysis showed the *gBRCA^MUT^* status of the serous OC patients was not associated with their PFS; instead, a nuclear expression of Ki-67% over 50% of the malignant cells appeared to be an independent predictor for a shorter PFS.

Mounting studies have shown that the prevalence of *gBRCA^MUT^* in ovarian cancer patients varies across different populations, ranging from 13.8% in Americans to 40% in Ashkenazi Jews (16–18). A nationwide multi-center study carried out by Wu et al. revealed the prevalence of *gBRCA^MUT^* in Chinese OC patients was 28.5% (7), which is comparable to our result. Interestingly, the top recurrently detected mutations varies between different studies, including the *BRCA1*:c.1081del, c.964del, c.3770_3771del, c.2371_2372del and c.5470_5477del *etc.* (12, 19–21); this is probably caused by the different ethnic origins and geographic locations of the included patients. Several studies have reported the *BRCA1*:c.5470_5477del as the most frequently detected mutation in Chinese OC patients; of note, the patients enrolled in these studies were mainly from northern China (13, 20, 22), and studies on the southern Chinese revealed different top mutations (12, 19, 21). In this study, almost all the patients are from Henan, the central region of China and the origin of Chinese civilization.

Predisposition of *gBRCA^MUT^* is known to promote the early-onset of breast and ovarian cancer (23). In our cohort, this phenomenon is more evident in the *gBRCA1^MUT^* carriers but not the *gBRCA2^MUT^* carriers of serous OC patients (median age: 51 *vs* 55; *p* = 0.0055) and to a much less extent in the *BRCA^MUT^* carriers in general as compared to the *BRCA^WT^* patients (median age: 52 *vs* 54; *p* = 0.0252). To our surprise, about 6.6% (35/530) of *BRCA^WT^* patients developed the disease before 40, in which only one presents with a clear family history, suggesting other unclarified risk factors contributing to their early disease on-set. In addition, the *BRCA^MUT^* carriers appeared to have the first peak of incidence from 40 to 44 years old, suggesting the *BRCA^MUT^* carriers should carry out preventative screening from 40.

STR analysis supports *BRCA1*:c.5470_5477del as the founder mutation in our OC cohort, but a validation of this finding in a larger cohort, maybe a nation-wide multi-centered study, could make the conclusion more solid. The conserved region from chr17:42627978 to 43223568 was only partially shared by *BRCA1*:c.981_982del carriers, which is probably caused by genomic rearrangement or other aberrations that occurred during the course of heredity. Recently, *BRCA1*:c.5470_5477del was also revealed as a founder mutation in a cohort of 9505 Han breast cancer patients, which made our conclusion more solid (24). Of note, studies carried out in the southern part of China identified different founder mutations, *i.e.*, *BRCA1*:1081del (12). A non-negligible limitation in all these studies, including this one, could be that only the coding regions of *BRCA1/2* and the flanking splice sites were sequenced, which missed the pathogenic aberrations in introns. For example, *BRCA1*:c.442-22_442-13del has been identified as an ancient founder mutation in the patients from the southern part of China (25).

Currently the first-line therapy against advanced ovarian cancer is maximal cytoreductive surgical debulking followed by chemotherapy with carboplatin–paclitaxel regimen (26). In this study, no difference in PFS was observed if the patients received adjuvant chemotherapy before surgery. Two independent studies showed neoadjuvant chemotherapy followed by surgery was not inferior to surgery followed by chemotherapy and with less postoperative adverse events (27, 28); however, a national cancer database study showed a superior overall survival in patients with primary surgery *versus* neoadjuvant chemotherapy (29). Surgery first or chemo first? There’s still not a conclusive answer at this point.

The prognosis of *BRCA^MUT^* carriers was shown better than *BRCA^WT^* carriers (30, 31), but we didn’t observe significant PFS benefit in *gBRCA^MUT^* carriers. This result is consistent with a recent study performed by You et al. that the benefit in PFS was only observed when the somatic *BRCA^MUT^* was also included into the analysis (22). Ki-67, a well-established biomarker of cellular proliferation, is frequently used in routine clinical workflow (32). Previous study showed Ki-67 expression was related to the overall survival of OC patients (33); here, our results confirmed that an expression of Ki-67 ≥50% indicated a shorter PFS. The efficiency of Ki-67 expression in predicting the prognostics of OC patients still needs improvement, and this could be addressed as the implication of high throughput sequencing based clinical tests are becoming increasingly prevalent.

In conclusion, our study reveals 28.3% of Henan OC patients are *gBRCA^MUT^* carriers, and *BRCA1*:c.5470_5477del is a founder mutation in Henan population. A nation-wide large cohort study may help us to understand the founder effect of *BRCA* mutations in Chinese and design a cost-effective screening test for the high-risk population.

## Data Availability Statement

The data presented in the study are deposited in the Genome Sequence Archive repository (https://bigd.big.ac.cn/gsa/), accession number PRJCA004762.

## Ethics Statement

The studies involving human participants were reviewed and approved by the ethics committee of Henan Cancer Hospital. The patients provided their written informed consent to participate in this study.

## Author Contributions

JL and YG conceived and designed the study. SH and JL analyzed the data and drafted the manuscript with the support from YG and HL. XW and CZ performed NGS test and bioinformatic analysis. YL and LW evaluated the clinical outcome of the patients. JM, BW, YW, and QX analyzed the IHC staining and were responsible for the pathological diagnosis. YG and HL supervised the project. All authors read and approved the submitted manuscript. All authors contributed to the article and approved the submitted version.

## Funding

This work was financially supported by the funding from Major public welfare projects in Henan Province (grant number: 201300310400) and Henan science and technology project (grant number: 212102310675). JL was also supported by the Henan provincial young researcher program. National Natural Science Foundation of China, 81802779 to JL; Henan Provincial Health Commission, SBGJ202002020 to JL; and Henan Science and Technology Project, 212102310675 to JL.

## Conflict of Interest

The authors declare that the research was conducted in the absence of any commercial or financial relationships that could be construed as a potential conflict of interest.
