# Corrigendum: Identification of *BRCA1*:c.5470_5477del as a Founder Mutation in Chinese Ovarian Cancer Patients

**DOI:** 10.3389/fonc.2021.840551

**Published:** 2022-01-14

**Authors:** Jun Li, Sile Han, Cuiyun Zhang, Yanlin Luo, Li Wang, Ping Wang, Yi Wang, Qingxin Xia, Xiaoyan Wang, Bing Wei, Jie Ma, Hongle Li, Yongjun Guo

**Affiliations:** ^1^ Department of Molecular Pathology, The Affiliated Cancer Hospital of Zhengzhou University and Henan Cancer Hospital, Zhengzhou, China; ^2^ Henan Key Laboratory of Molecular Pathology, Zhengzhou, China; ^3^ Department of Gynecologic Oncology, The Affiliated Cancer Hospital of Zhengzhou University and Henan Cancer Hospital, Zhengzhou, China; ^4^ Department of Pathophysiology, School of Basic Medical Science, Zhengzhou University, Zhengzhou, China; ^5^ Department of Pathology, The Affiliated Cancer Hospital of Zhengzhou University and Henan Cancer Hospital, Zhengzhou, China

**Keywords:** BRCA1/2, haplotype analysis, ovarian cancer, founder mutation, Chinese

In the original article, we neglected to include the funder National Natural Science Foundation of China, 81802779 to Jun Li; Henan Provincial Health Commission, SBGJ202002020 to Jun Li; and Henan Science and Technology Project, 212102310675 to Jun Li.

The authors apologize for this error and state that this does not change the scientific conclusions of the article in any way. The original article has been updated.

## Publisher’s Note

All claims expressed in this article are solely those of the authors and do not necessarily represent those of their affiliated organizations, or those of the publisher, the editors and the reviewers. Any product that may be evaluated in this article, or claim that may be made by its manufacturer, is not guaranteed or endorsed by the publisher.

