# Supplementary material for: Identification of BRCA1:c.5470_5477del as a Founder Mutation in Chinese Ovarian Cancer Patients
Source: Front Oncol. 2021 May 11;11:655709. doi: 10.3389/fonc.2021.655709 (PMC8148338; doi:10.3389/fonc.2021.655709)
Supplement: Supplementary file 1 [file DataSheet_1.docx]

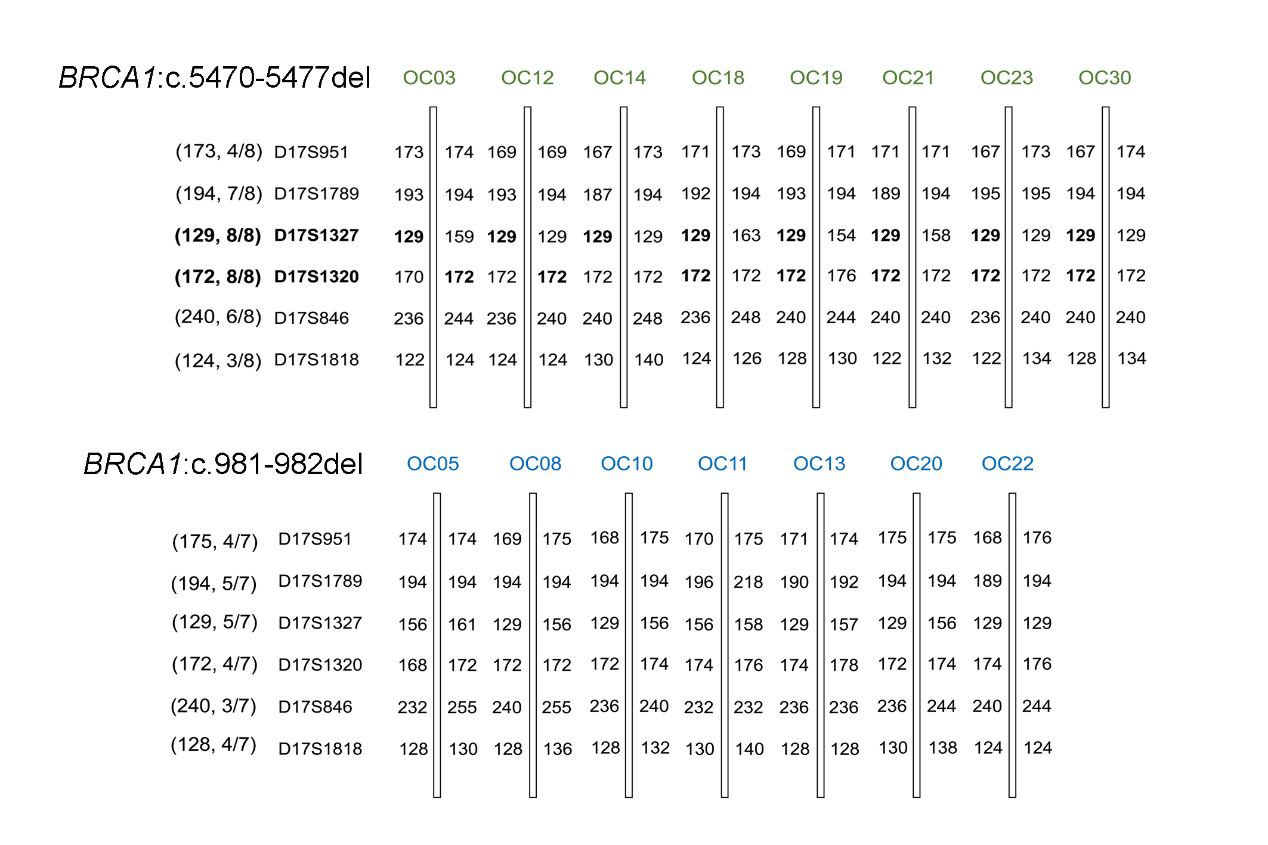


Supplementary figure 1. Haplotype analysis of STR markers flanking *BRCA1* across all 8 *BRCA1*:c.5470_5477del and 7 c.981_982del carriers.
