# Supplementary material for: Identification of BRCA1:c.5470_5477del as a Founder Mutation in Chinese Ovarian Cancer Patients
Source: Front Oncol. 2021 May 11;11:655709. doi: 10.3389/fonc.2021.655709 (PMC8148338; doi:10.3389/fonc.2021.655709)
Supplement: Supplementary file 2 [file Table_1.docx]

| **Primer name** | **Sequence (5' to 3')** |
| --- | --- |
| D17S951F | GGCCTCCCAAACTGCTT |
| D17S951R | TCTACCCCGATGAGCCA |
| D17S1789F | ATTGNCCTGGCTTCTG |
| D17S1789R | GGCTGGAGCAGGGACT |
| D17S846F | TGCATACCTGTACTACTTCAG |
| D17S846R | TCCTTTGTTGCAGATTTCTTC |
| D17S1818F | CATAGGTATGTTCAGAAATGTGA |
| D17S1818R | TGCCTACTGGAAACCAGA |
| D17S1327F | CTAAGGAGGTTTCTCTGGAC |
| D17S1327R | TTCACAACTCAAGGTAAGATAGG |
| D17S1320F | ACTTTCCAGAAAATCTCTGCTC |
| D17S1320R | CCACGTCTTTTCTGTGTTCC |

Supplementary Table1. Primer sequences used for detecting 6 STR markers flanking *BRCA1*.
